# When healthy classrooms hurt: how reduced bullying isolates victimized youth through altered friendship and status dynamics

**DOI:** 10.1186/s40359-026-04141-0

**Published:** 2026-02-18

**Authors:** Tamás Hoffmann, Bence Basa, Katalin N. Kollár

**Affiliations:** 1 Institute of Psychology, ELTE, Eotvos Lorand University, 1064, Budapest, Izabella utca 64, Budapest, Hungary; 2 Doctoral School of Psychology, Institute of Psychology, ELTE, Eotvos Lorand University, Izabella u. 64, Budapest, 1064 Hungary; 3 Independent Researcher, Budapest, Hungary

**Keywords:** Bullying, Friendships, Acceptance, Rejection, Healthy context paradox

## Abstract

The healthy context paradox—the phenomenon that victimized youths show poorer adjustment in classrooms with lower levels of bullying—has been documented primarily for psychological outcomes but remains less explored with respect to peer social dynamics. The present study examined whether this pattern is also observable in victims’ peer acceptance, and rejection beyond their friendship network. Using sociometric data from 915 Hungarian students (aged 11–18) across 40 classrooms, we estimated multilevel models to test whether classroom-level victimization norms were associated with variation in the relationship between individual victimization and social outcomes. Results indicated that victimized students in classrooms characterized by lower victimization norms reported fewer mutual friendships, lower acceptance, and higher rejection compared to victimized students in classrooms with higher victimization norms. These findings suggest that associations between victimization and peer social standing differ systematically across classroom contexts. The study highlights the importance of considering classroom-level nomination patterns when interpreting sociometric indicators and discusses implications for research on peer relations in low-bullying environments.

**Supplementary Information:**

The online version contains supplementary material available at 10.1186/s40359-026-04141-0.

## Introduction

Bullying is a pervasive negative phenomenon that has been extensively studied in the literature, with research examining bullying and defending behaviors as well as consequences for victims [1–3]. Victimized youth exhibit poorer overall adjustment [4] and face detrimental psychological outcomes, including heightened depressive symptoms [5, 6], anxiety [7], and an increased risk of self-injury [8]. Consequently, intervention efforts have sought to reduce bullying prevalence. Given its group-based nature, these interventions typically adopt a whole-classroom or whole-school approach, fostering a sense of belonging and promoting an anti-bullying mindset. Such efforts encourage children to engage in bully- or victim focused defending behaviors toward victims [9] and, crucially, reduce the likelihood of reinforcing bullies by enhancing their social status which is a key motivator for aggressors [10–12].

Recent studies have identified a counterintuitive phenomenon: although successful interventions generally enhance overall well-being in classrooms [13], students who experience bullying in these more favorable environments may exhibit stronger symptoms, poorer adjustment and be less accepted by their peers compared to those in less supportive settings [14–16]. This process, termed the healthy context paradox, has become a key focus for recent research and may have important implications for intervention efforts [15, 17, 18]. The phenomenon is typically examined by relating individual-level adjustment outcomes to classroom-level bullying norms. These norms are most often operationalized as descriptive norms, defined as the average level of bullying or victimization within a classroom, but can also be conceptualized as popularity norms, reflecting the association between bullying and peer popularity at the classroom level [15, 19].

Several theoretical explanations for this paradox have been proposed drawing on social comparison theory, social misfit theory, defending inflation theory, and—most importantly for the present study—relational dynamics between victims and non-victims [17].

Social comparison theory [20] posits that individuals evaluate their social standing by comparing themselves to others. In healthier contexts (i.e. where bullying is less prevalent), victims may fare worse because they encounter fewer maladjusted peers and more well-adjusted non-victims, amplifying upward social comparisons. These comparisons can inflate self-perceptions, exacerbating feelings of inadequacy [5]. Similarly, social misfit theory suggests that children who deviate from peer-group norms struggle with adjustment and friendship formation [21]. If the victim is the only or one of the few victims in an otherwise well-adjusted classroom, the student’s situation may be viewed more negatively by peers, and this may lead to fewer opportunities for them to form friendships and a general sense of belonging to the group which may foster feelings of isolation [22]. Both theories further imply an internalized locus of control. In predominantly healthy environments, the scarcity of other victims may lead bullied children to attribute victimization to personal flaw instead of correctly identifying it as a group-process which can foster self-blame [23, 24]. Defending inflation theory offers an alternative perspective, proposing that in positive environments, many children attempt defending behaviors to conform to group norms. However, effective defending typically requires stable social status and strong interpersonal skills [25]. When less socially adept peers intervene, their efforts may be ineffective, inadvertently worsening victims’ problems [17]. Together, these perspectives highlight important individual-level processes that affect adjustment but offer limited insight into how victims’ positions within peer networks and status hierarchies may differ across classroom contexts.

Several studies suggest that the negative effects associated with the healthy context paradox are linked to victims’ *peer relationships*, particularly their friendships in classrooms with different norm structures [15, 26]. This line of work suggests that the healthy context paradox may be expressed not only in psychological adjustment, but also in victims’ social integration within the peer group which may be a key factor in the process that leads to poorer adjustment. Beyond individual-level theories outlined above, network and sociometric perspectives highlight structural barriers to friendship formation. They posit that selection and influence, two important processes for friendship formation and maintenance may underlie the healthy context paradox [26]. Selection is the process by which peers with similar interests and social standing are more likely to form friendships, while influence describes the convergence of friends’ behaviors and attitudes over time [26–28]. Recent studies suggest that victims in positive peer environments may be at a greater disadvantage in finding friends [5]. While shared victimization can facilitate friendship formation among bullied youth in less supportive environments [15], this mechanism is disrupted in healthier contexts due to the relative scarcity of similarly affected peers. Additionally, non-victimized children may be less likely to befriend victims due to concerns about status contagion—the perceived risk of being targeted themselves [28]. Although victim-victim friendships tend to be of lower quality [29], friendships have been consistently shown to mitigate the detrimental effects of victimization [6]. Paradoxically, students in healthier environments face greater risks because friendship opportunities diminish. Pan et al. [5] found that victimized children in low-bullying classrooms receive fewer friendship nominations, and that the lack of friendships longitudinally predicts poorer psychological adjustment.

While another field of research of children’s relationships, sociometric studies, have established robust links between victimization and peer status generally [6, 30], these methods remain less studied in research on the healthy context paradox. Prior work primarily focused on adjustment variables (e.g., depression, self-esteem) to identify the paradox [5, 15], leaving its social mechanisms underexplored. Though sociometry has been applied to bullying research broadly (e.g., 6), its potential to validate the paradox through classroom-level acceptance/rejection patterns and clarify how victimized youth’s social position deteriorates in ‘healthier’ contexts remains untapped. As a result, it remains unclear whether the healthy context paradox is also evident in sociometric indicators of peer relationships.

### The current study

Growing evidence for the healthy context paradox highlights its importance for school-based anti-bullying interventions and a more nuanced understanding of bullying. This study addresses a research gap by examining associations between victimization and peer status, extending prior work on friendships to include peer acceptance and rejection across classrooms differing in victimization norms.

Building on existing findings, we hypothesized that the association between individual victimization and peer status would differ across classrooms with lower versus higher levels of victimization. Specifically, given evidence that the friendship patterns of victimized youth are negatively affected in low-victimization contexts, we expected higher rejection and lower acceptance among victimized students in classrooms characterized by lower overall victimization.

## Method

### Procedure

Data were collected in 2024 using eSzocMet, a free online sociometric tool designed by the study authors to measure classroom peer dynamics. The software streamlines sociometric assessments, an approach that is widely used by Hungarian school psychologists, greatly enhancing time efficiency in regular screenings. The peer-nomination questionnaire was disseminated via eSzocMet’s social media platform to school psychologists conducting sociometric testing.Due to the recruitment method, most participating practitioners were already familiar with the tool. Although step-by-step guides and e-mail support were provided, no issues were reported during data collection. Practitioners accessed the full dataset, while the software generated anonymized research data for this study. Data were screened for errors (e.g., omitted questions) and missing responses; only classrooms with 100% participation and complete questionnaires were retained.^1^

The anonymization process, the legal and ethical handling of the data and the procedure of testing are in accordance with the General Data Protection Regulation of the European Union, has been developed under the supervision of technical and legal experts and is based on the guidelines given by the Hungarian National Authority for Data Protection and Freedom of Information specifically for this software. The entire process was reviewed and approved by the Ethical Board of Lorand Eötvös University in ethical permission no. 2023/1.

### Sample

The study included 915 students (43% girls) across 40 classrooms (M = 24.5; Min = 8, Max = 36; SD = 5.81). To ensure adequate statistical power and examine potential age-related variations in the healthy context paradox, we intentionally sampled from both upper elementary (age 11–14) and secondary school (age 15–18) populations (see Table 1 for distribution).

**Table 1:** Distribution of grade levels

Grade level	Number of classrooms in the dataset
6th grade	8
7th grade	5
8th grade	7
9th grade	9
10th grade	3
11th grade	2
12th grade	2

## Measures

### Acceptance and rejection

Acceptance and rejection were assessed through two unlimited peer-nomination items; ‘Who do you like most?’ and ‘Who do you like least?’ [31]. Consistent with Gommans and Cillessen’s [32] suggestions, the software capped processed nominations at 10 per item to mitigate indiscriminate responding while preserving natural nomination patterns.

### Friendship patterns

Friendships were evaluated using Mérei’s tripartite peer-nomination system, the standard tool among Hungarian school psychologists. This method combines one ‘best friend’ question with two contextual scenario-based items (in the case of this study: ’a peer you can turn to’ and ’those you wish to keep in touch with after finishing school’), each limited to 3 peer-nomination choices. Mutual nominations were recorded across all three items, allowing for multiple reciprocal ties. Mérei’s method was selected instead of a single unlimited nomination friendship question because it reflects the established sociometric practice in Hungarian schools and aligns with the training and assessment routines of the professionals who administered the study [33].

### Victimization

Bullying roles (victim, bully, defender) were assessed via three peer-nomination items, each prefaced by a definition of bullying and limited to three nominations. Self-nominations were allowed in these questions to capture both peer-perceived and self-identified victimization—a dual-method approach addressing known divergence between these measures [34] and an important tool for practitioners (an English translation of the questions used are available in Appendix 1).

### Peer-context

Following Laninga Wijnen et al. [15] and other researchers, we operationalized classroom-level victimization norms using the mean victimization score within each classroom. This classroom-level mean reflects the overall salience of victimization nominations within the classroom rather than the number of distinct victims.

### Analytic plan

We tested our hypotheses using multilevel linear modeling in R (v4.4.0) to account for nested student-classroom data structures. Three parallel models examined friendship, acceptance, and rejection as dependent variables, with predictors including individual victimization, gender, classroom grade level, descriptive norm for victimization within each classroom, and, as the main focus of this study, an interaction term between individual victimization and classroom norms. All models were planned to include random intercepts and random slopes allowing the assessment of whether the strength of the association between individual victimization and social outcomes varies across classroom contexts, while controlling for individual-level characteristics.

To enable cross-classroom comparability, raw nomination counts were first converted to proportion scores by dividing by classroom size and then rescaled by multiplying by 25 to reflect scores for the median class size of the dataset. While mathematically equivalent to proportion-based approaches, this scaling facilitates more intuitive interpretation of regression coefficients in the estimated models.

Nomination limits constrain absolute counts in the friendship questions and empirical evidence suggests that network size only weakly affects mean friendship formation [35] which was also true in this dataset (correlation between class size and average number of friendships was *r*=.16, *p*=.32) therefore raw mutual friendship counts were used for the analyses. Further, victimization, acceptance and rejection scores were classroom-mean-centered to optimize model convergence and interaction testing.

## Results

Table 2 presents correlations among the study variables at the individual level, whereas Table 3 presents correlations aggregated at the classroom level. At the individual level, victimization was negatively associated with mutual friendships and acceptance and positively associated with rejection. Acceptance and rejection were negatively correlated, while acceptance was positively correlated with mutual friendships.

At the classroom level, strong associations emerged between victimization, acceptance, and rejection, whereas these variables showed no association with the number of mutual friendships. This pattern was examined further in the model assumptions section.

**Table 2:** Correlations between the investigated variables (individual level)

Variable	M	SD	1	2	3
1. Victimization	1.10	2.47			
2. Number of mutual friendships	2.15	1.19	− 0.23** [-0.29, − 0.16]		
3. Acceptance	2.15	1.67	− 0.24**	0.57**	
			[-0.30, − 0.17]	[0.52, 0.61]	
4. Rejection	2.63	1.71	0.38**	− 0.24**	− 0.30**
			[0.32, 0.43]	[-0.30, − 0.17]	[-0.36, − 0.24]

* indicates *p* < .05. ** indicates *p* < .01

### Model assumptions

Prior to hypothesis testing, we rigorously evaluated model assumptions. Variance inflation factors (all < 5.5) and tolerance statistics (all > 1.5) confirmed the absence of problematic multicollinearity. However, visual inspection of residual plots and Shapiro-Wilk tests indicated moderate violations of normality and homoscedasticity for the acceptance and rejection models, likely attributable to the right-skewed distribution of nominations (acceptance: M = 2.2, SD = 1.6; rejection: M = 2.6, SD = 1.7). These variables also showed lower nomination frequencies than typically reported in sociometric literature (i.e.: Lafontana & Cillessen, 2015). To verify result stability, we re-estimated models using robust standard errors; all significant effects persisted or strengthened, confirming their reliability despite assumption violations.

Likely due also to the skewed distribution of nomination data for the acceptance and rejection models, convergence issues prevented estimation of random effects. Consequently, these analyses employed multiple linear regressions without random effects, while retaining all other model specifications (predictors, controls, and interaction terms). For the friendship outcome, the intraclass correlation coefficient (ICC) from the null model was 0.07, indicating modest but non-negligible classroom-level clustering. For the acceptance and rejection models, ICC values were below 0.03, indicating minimal between-classroom variance and supporting the use of single-level regression models.

Further, initial models revealed unexpected positive associations between classroom-level victimization norms and rejection and acceptance scores (as evidenced in Table 3). Investigation showed that descriptive norms for victimization were highly correlated with the general tendency to nominate across all items within classrooms (*r* > .7), suggesting a potential confounding effect; classrooms with higher overall nomination activity might artifactually appear to have stronger victimization norms. To account for this, we introduced a classroom-level control for general nomination tendency—calculated as the average of all proportion-scaled peer nomination variables, including behavioral descriptors that were not directly analyzed in the present study (i.e.: scaled bullying and defender nominations). This adjustment ensured that observed effects of victimization norms reflected specific social processes rather than generalized response tendencies.

**Table 3:** Correlations between the investigated variables (classroom level)

Variable	M	SD	1	2	3
1. Victimization	1.36	0.8			
2. Number of mutual friendships	2.14	0.35	− 0.02 [-0.38, − 0.25]		
3. Acceptance	2.57	1.07	0.82**	− 0.01	
			[0.68, 0.90]	[-0.32, 0.30]	
4. Rejection	2.03	0.79	0.84**	0.07	0.86**
			[0.71, − 0.91]	[-0.24, 0.38]	[0.75, 0.92]

* indicates *p* < .05. ** indicates *p* < .01

### Hypothesis testing

#### Friendships

**Fig. 1:**
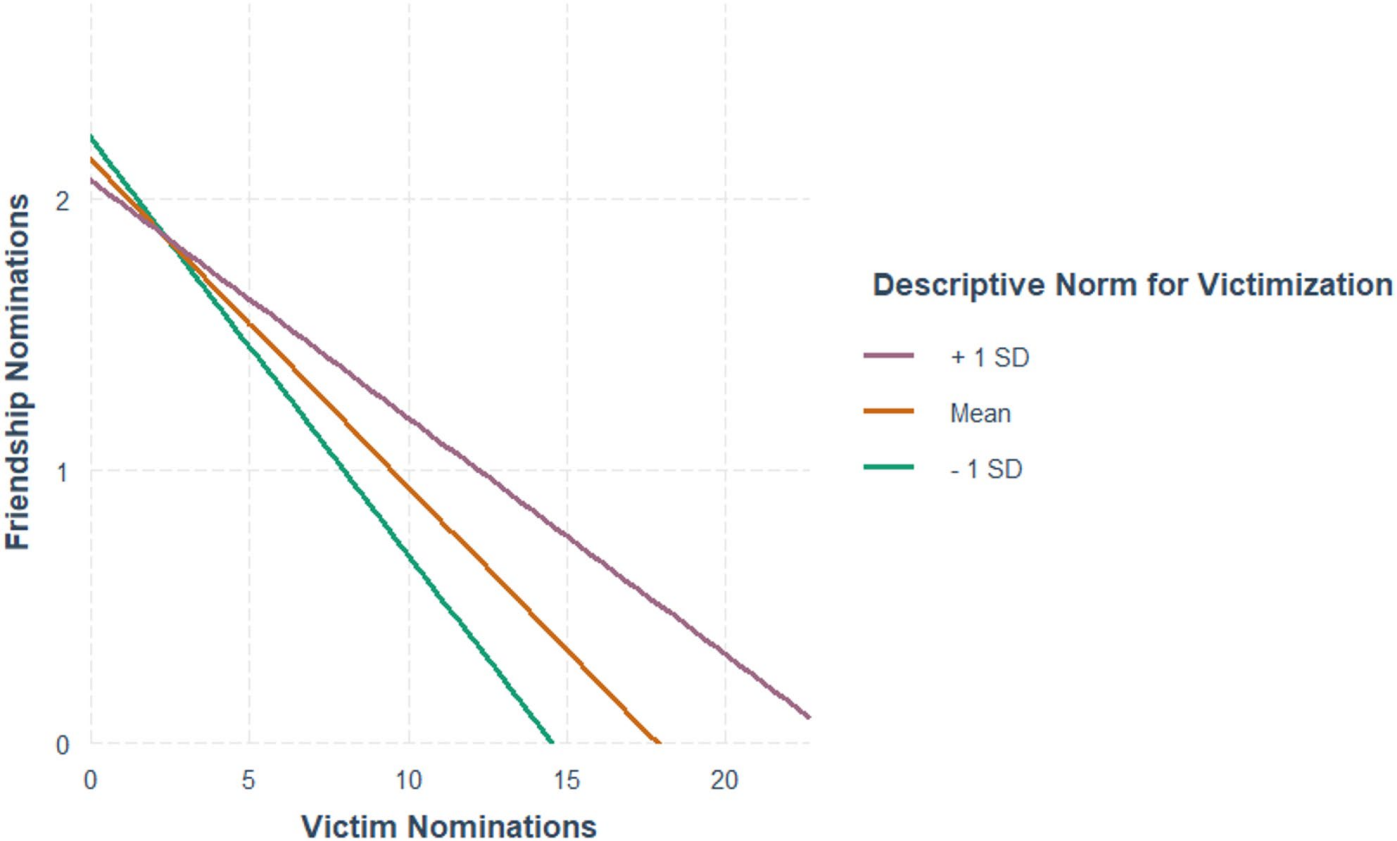
Friendships regressed on victimization nominations by descriptive norms

The multilevel model for mutual friendships yielded three key findings (Table 4). First, individual victimization significantly predicted fewer mutual friendships corresponding to approximately one fewer friendship per five victimization nominations. Second, the interaction between individual victimization and classroom victimization norms was significant. As illustrated in Fig. 1, simple slope analyses indicated that the strength of the association between victimization and friendships differed across levels of classroom victimization norms, with pairwise comparisons indicating a smaller negative association at higher levels of classroom victimization norms (Δb = − 0.10, SE = 0.04, *p* < .05).

Third, gender, class level, and the main effect of classroom victimization norms were not statistically significant. Marginal R² indicated that fixed effects explained approximately 6% of the variance, while conditional R² showed that including random effects increased explained variance to 10%.

#### Acceptance

**Fig. 2:**
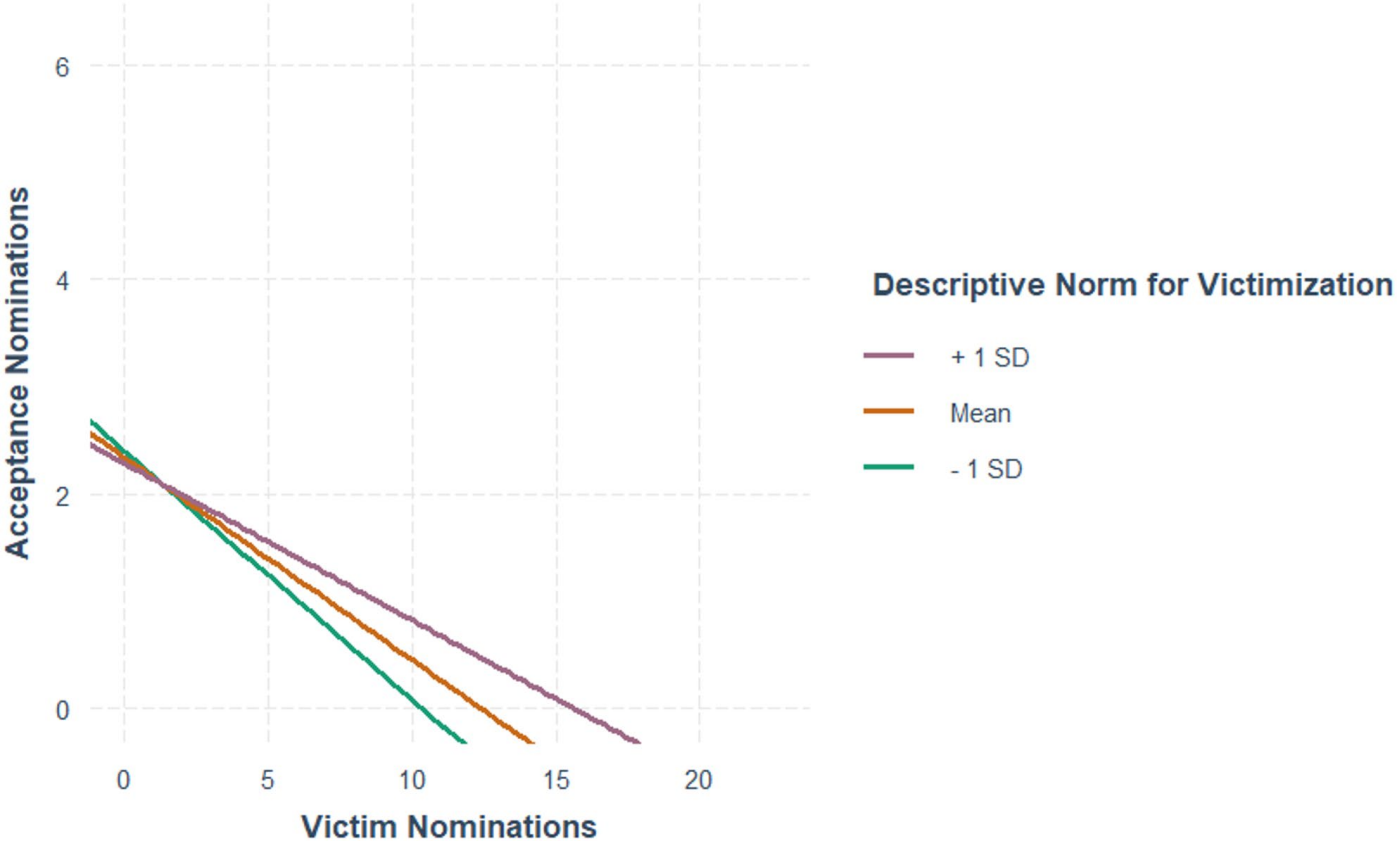
Scaled acceptance nominations regressed on victimization nominations by descriptive norms

The multiple linear regression model for peer acceptance (Table 5) indicated that individual victimization significantly predicted lower acceptance, corresponding to an approximately one-point decrease in acceptance scores for each four-unit increase in victimization. Second, the interaction between individual victimization and classroom victimization norms was significant. As shown in Fig. 2, simple slope analyses indicated that the strength of the negative association between victimization and acceptance differed across levels of classroom victimization norms, with pairwise comparisons showing a smaller negative association at higher levels of classroom victimization norms (Δb = 0.13, SE = 0.06, *p* < .05).

**Table 4:** Regression model for friendship nominations

Predictors (level 1 then level 2)	Estimates	CI	*p*	
(Intercept)	2.07	1.29–2.85	**< 0.001**	
Victimization	-0.18	-0.25 – -0.11	**< 0.001**	
Gender (Boys = 0)	0.11	-0.05–0.27	0.166	
Descriptive norm (victimization)	-0.12	-0.40–0.16	0.416	
Grade level	-0.02	-0.09–0.04	0.460	
Global nomination rate	0.11	-0.14–0.36	0.395	
Victimization × Descriptive norm (victimization)	0.05	0.01–0.09	**0.013**	
**Random Effects**				
σ^2^ = 1.28		τ_00_ = 0.06		ICC = 0.06
Marginal R^2^ / Conditional R^2^ = 0.063 / 0.106				

The classroom-level global nomination rate was positively associated with acceptance scores, indicating that classrooms with higher overall nomination activity tended to yield higher acceptance scores. Gender, class level, and the main effect of classroom victimization norms were not statistically significant when included alongside other predictors. The model explained approximately 20% of the total variance.

#### Rejection

**Fig. 3:**
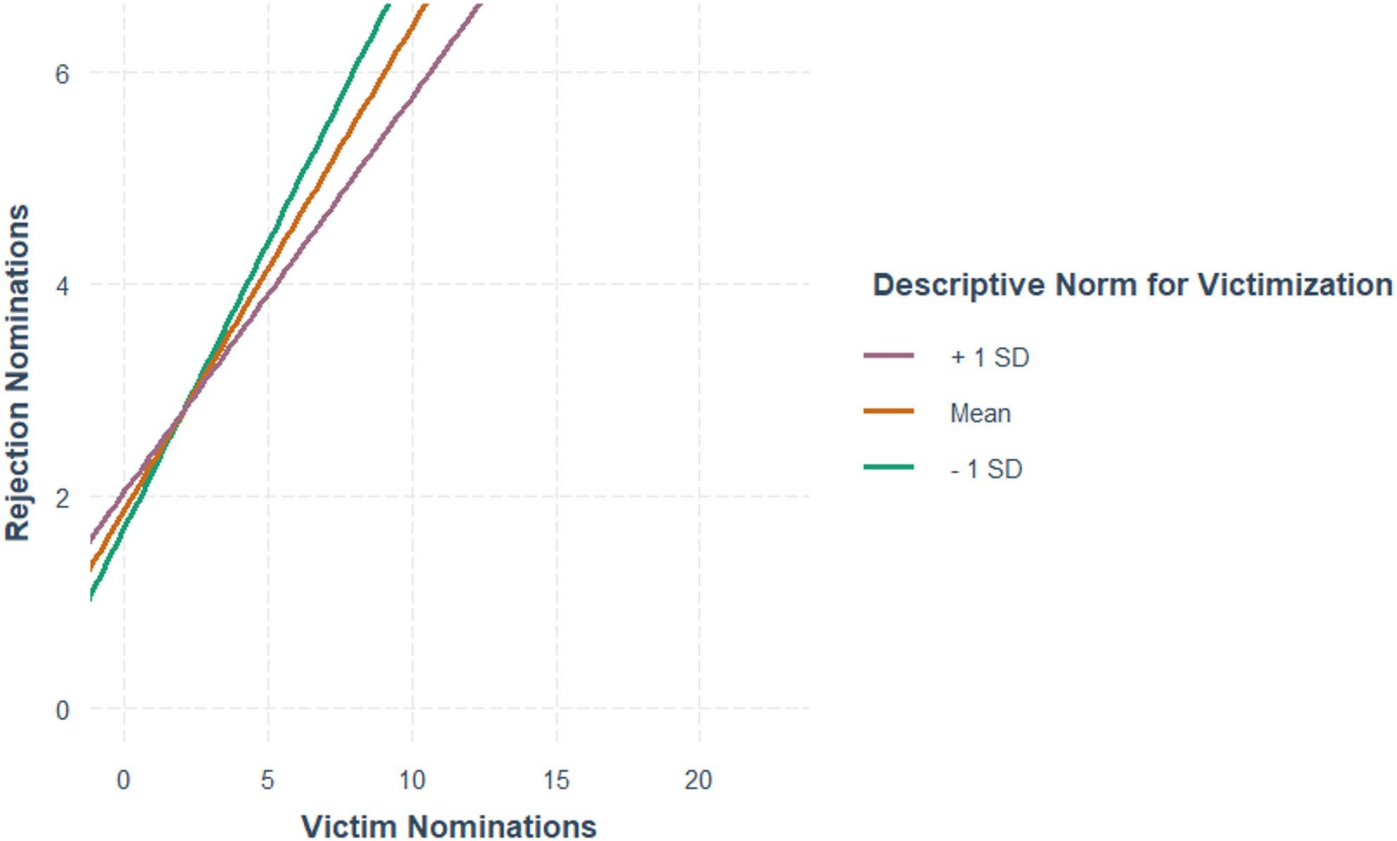
Scaled rejection nominations regressed on victimization nominations by descriptive norms

The multiple linear regression model for peer rejection (Table 6) showed that individual victimization significantly predicted higher rejection scores, corresponding to an increase of more than half a point in rejection for each unit increase in victimization. The interaction between individual victimization and classroom victimization norms was also significant. As illustrated in Fig. 3, simple slope analyses indicated that the strength of the positive association between victimization and rejection differed across levels of classroom victimization norms, with pairwise comparisons showing a smaller positive association at higher levels of classroom victimization norms (Δb = 0.25, SE = 0.09, *p* < .01).

Rejection was positively associated with the classroom-level global nomination rate. Gender, class level, and the main effect of classroom victimization norms were not statistically significant when included alongside other predictors. The model explained approximately 18% of variance.

**Table 5:** Acceptance regressed on victimization nominations by descriptive norms

Predictors (level 1 then level 2)	Estimates	CI	*p*
(Intercept)	0.09	-0.82–1.00	0.845
Victimization	-0.27	-0.37 – -0.16	< 0.001
Gender (Boys = 0)	0.02	-0.22–0.27	0.847
Descriptive norm (victimization)	-0.09	-0.41–0.24	0.595
Grade level	-0.02	-0.09–0.05	0.554
Global nomination rate	1.11	0.82–1.40	< 0.001
Victimization ×Descriptive norm (victimization)	0.06	0.00–0.13	0.044
R^2^ / adjusted R^2^	0.208 / 0.202		

## Discussion

This study examined whether patterns consistent with the healthy context paradox—previously observed for adjustment outcomes [15]—are also observable in aspects of social standing. Drawing from theories of social comparison and locus of control, we hypothesized that victimization would be associated with increased rejection, decreased acceptance and number of mutual friendships in classrooms with lower descriptive norms for victimization. 

**Table 6:** Rejection regressed on victimization nominations by descriptive norms

Predictors (level 1 then level 2)	Estimates	CI	*p*
(Intercept)	-0.10	-1.59–1.38	0.892
Victimization	0.61	0.45–0.76	**< 0.001**
Gender (Boys = 0)	0.16	-0.19–0.51	0.374
Descriptive norm (victimization)	0.26	-0.25–0.77	0.323
Grade level	0.00	-0.10–0.11	0.942
Global nomination rate	0.67	0.13–1.22	**0.015**
Victimization ×Descriptive norm(victimization)	-0.13	-0.22 – -0.04	**0.007**
Observations	915		
R^2^ / R^2^ adjusted	0.183 / 0.178		

The results were consistent with these hypotheses, with all three interaction terms reaching statistical significance. These findings align with the healthy context paradox framework, indicating that associations between victimization and social standing are more negative in classrooms with lower levels of victimization. While previous research has established this pattern for depressive symptoms [5, 18], friendships [28] and adjustment [15], the present study extends these findings to sociometric indicators of peer status, specifically peer acceptance and rejection beyond friendship ties. In doing so, the study provides additional support for the healthy context paradox and suggests that its implications extend beyond individual well-being to victims’ social positioning within the peer group. However, the extent to which lower sociometric status is associated with poorer adjustment in healthier classroom contexts remains an open question.

One possible interpretation of these findings is that victims’ social marginalization in classrooms with lower victimization norms may reflect multiple interrelated relational processes. From a similarity perspective [26], friendship formation among victimized youth may be more constrained in such environments because fewer peers share similar victimization experiences. In addition, social misfit theory suggests that children who deviate from prevailing peer norms may be more vulnerable to rejection and less likely to receive acceptance [21]. Together, these perspectives suggest that differences in victims’ social standing across classroom contexts may be linked to broader relational dynamics. An alternative interpretation is also plausible. For example, prior research suggests that upward social comparisons in classrooms with lower levels of victimization may be associated with poorer psychological adjustment among victims [5]. Such psychological distress could, in turn, be related to differences in victims’ social positioning, including friendship formation and peer status. While the present study cannot address the temporal ordering of these processes due to its cross-sectional design, the observed patterns highlight the need for longitudinal research to clarify the directionality of these associations.

The analyses also highlighted classroom-level variation in general nomination tendency (range = 2–6 nominations), which was associated with both sociometric outcome variables and key predictors in the models. This observation speaks to ongoing methodological discussions regarding sociometric standardization [36, 37]. Whereas traditional approaches such as grand-mean-centering or standardization remove between-classroom variation, the present results suggest that classroom-specific nomination tendencies may meaningfully shape sociometric indicators and therefore warrant explicit consideration in studies using descriptive norms. By controlling for classroom nomination rates, we preserved natural between-class variation while isolating within-classroom effects. This approach parallels Terry’s [38] individual-level method at the group level, who argued that nominator specific factors (i.e. selectivity) should be included in the analysis of sociometric data. Though further research must validate this, our results preliminarily suggest that nomination tendencies may confound studies using descriptive norms unless explicitly modeled.

### Strengths and limitations

#### Strengths

The study has notable strengths. First, it is the first to simultaneously examine friendship networks and sociometric status in the context of the healthy context paradox. By demonstrating that victims in low-victimization classrooms face compounded social risks; fewer friends, lower acceptance, and higher rejection, we extend the paradox beyond psychological outcomes to interpersonal dynamics.

Second, the integration of friendship, bullying and sociometric data addresses a gap. Most studies analyze these domains separately, however the interplay between these dimensions reveals may have important research and practical implications that can only be studied with the simultaneous use of these variables [39, 40].

Third, our handling of nomination tendency may draw attention to an important factor in sociometric research. By identifying and controlling for classroom-level variation in nomination rates we can isolate more robust estimates, especially in studies using descriptive norms. This finding adds to the literature of standardizing sociometric results which have been an ongoing debate since the introduction of the currently used methods [31, 36, 38].

Fourth, the study expands sociometric research’s cultural scope. Most literature derives from Western European/North American contexts; our Hungarian sample provides needed diversity.

#### Limitations

The study has several limitations to consider.

First, the nomination rates observed in this study and other studies done on a Hungarian sample [41] is considerably lower than observed in other samples. The use of several nomination formats (limited and unlimited items) could have inadvertently introduced anchoring effects for unlimited nomination questions, making responders calibrate their nominations to the limit of three even if the limit did not apply to most of the questions which was clearly displayed in the software.

Additionally, the similarity between friendship and sociometric items may have confused some respondents, further suppressing nomination rates by the above procedures. These factors may limit generalizability to different contexts and may be an important area for future research as peer-nominated items on different yet intertwined aspects on children’s social lives (i.e.: friendships and acceptance) may require such questions to be included in the questionnaires which may interfere with each other.

Second, the cross-sectional design prevents causal interpretations regarding directionality. Although longitudinal studies have shown that victims in low-victimization classrooms experience declining adjustment [14, 17], our data cannot determine whether weakened social ties drive or result from this process; it only captures a pattern that aligns with the expectations of the healthy context paradox framework.

Third, the low nomination rates likely contributed to the multilevel models’ convergence issues for acceptance/rejection outcomes. Though mitigated in part by including classroom nomination tendency as a covariate, this prevented exploration of random effects of cross-classroom variance in victimization’s social consequences which was an original aim of the study.

Fourth Mérei’s method for friendship nomination is a long-standing tradition in Hungarian school psychology and was useful for building a larger database as professionals could use the results in their practice and were thereby more likely to participate. The methodology, however, does not align with established modern best practices in friendship-network research and likely inflated the number of mutual friendships detected [42].

## Conclusion

Consistent with the healthy context paradox, this study identified more negative associations between victimization and social standing in classrooms characterized by lower levels of victimization. While we cannot determine the direction of these effects, the findings open new questions for future research to explore.

### Practical implications

These findings may offer tentative insights for anti-bullying interventions. Although reducing classroom victimization appears to benefit the peer group as a whole, the present results suggest that some victims in such environments might experience increased social marginalization (fewer friendships, lower acceptance, higher rejection) compared to victims in classrooms with higher levels of victimization. Accordingly, it may be advisable for schools, educators, and school psychologists to monitor victims’ social relationships following the implementation of anti-bullying programs to ensure that the social well-being of these students is not inadvertently overlooked.

## Supplementary Information

Supplementary Material 1.

## Data Availability

The dataset is available at [https://github.com/hofical/Healthycontext](https:/github.com/hofical/Healthycontext).

## Footnotes

1 While the requirement of full classroom participation may appear stringent, only one classroom was excluded from the dataset due to incomplete responses. This high compliance rate reflects the established practice of sociometric testing in Hungarian schools, where these assessments are routinely administered by trusted professionals (head teachers and school psychologists) in educational settings. Within this cultural and professional context, student opt-outs occur infrequently.

## References

[1] Kochenderfer-Ladd B, Wardrop JL. Chronicity and instability of children’s peer victimization experiences as predictors of loneliness and social satisfaction trajectories. Child Dev. 2001;72(1):134–51. 10.1111/1467-8624.00270. 11280475 10.1111/1467-8624.00270

[2] Salmivalli C, Peets K. Bullying and victimization. In: Bukowski WM, Laursen B, Rubin KH, editors. Handbook of peer interactions, relationships, and groups. 2nd ed. Guilford Press; 2018. pp. 302–21.

[3] Veenstra R, Lindenberg S, Oldehinkel AJ, De Winter AF, Verhulst FC, Ormel J. Bullying and victimization in elementary schools: A comparison of bullies, victims, bully/victims, and uninvolved preadolescents. Dev Psychol. 2005;41(4):672–82. 10.1037/0012-1649.41.4.672. 16060813 10.1037/0012-1649.41.4.672

[4] Ladd GW, Troop-Gordon W. The role of chronic peer difficulties in the development of children’s psychological adjustment problems. Child Dev. 2003;74(5):1344–67. 10.1111/1467-8624.00611. 14552402 10.1111/1467-8624.00611

[5] Pan B, Li T, Ji L, Malamut S, Zhang W, Salmivalli C. Why does classroom-level victimization moderate the association between victimization and depressive symptoms? The healthy context paradox and two explanations. Child Dev. 2021;92(4):1836–54. 10.1111/cdev.13624. 34196997 10.1111/cdev.13624

[6] Salmivalli C, Isaacs J. Prospective relations among victimization, rejection, friendlessness, and children’s self- and peer-perceptions. Child Dev. 2005;76(6):1161–71. 10.1111/j.1467-8624.2005.00841.x. 16274432 10.1111/j.1467-8624.2005.00842.x

[7] Balluerka N, Aliri J, Goñi-Balentziaga O, Gorostiaga A. Association between bullying victimization, anxiety and depression in childhood and adolescence: the mediating effect of self-esteem. Revista De Psicodidáctica (English ed). 2023;28(1):26–34. 10.1016/j.psicoe.2022.11.001.

[8] Serafini G, Aguglia A, Amerio A, Canepa G, Adavastro G, Conigliaro C, Nebbia J, Franchi L, Flouri E, Amore M. The relationship between bullying victimization and perpetration and non-suicidal self-injury: A systematic review. Child Psychiatry Hum Dev. 2023;54(1):154–75. 10.1007/s10578-021-01231-5. 34435243 10.1007/s10578-021-01231-5 PMC9867675

[9] Menesini E, Salmivalli C. Bullying in schools: the state of knowledge and effective interventions. Psychol Health Med. 2017;22(sup1):240–53. 10.1080/13548506.2017.1279740. 28114811 10.1080/13548506.2017.1279740

[10] Andrews NCZ, McDowell H, Spadafora N, Dane AV. Using social network position to understand early adolescents’ power and dominance within a school context. School Psychol. 2022;37(6):445–54. 10.1037/spq0000445. 10.1037/spq0000445 34410798

[11] Veenstra R, Huitsing G. (2021). Social network approaches to bullying and victimization. In P. K. Smith & J. O. Norman, editors, *The Wiley Blackwell handbook of bullying* (pp. 127–143). Wiley. 10.1002/9781118482650.ch11

[12] Veenstra R, Lindenberg S, Zijlstra BJH, De Winter AF, Verhulst FC, Ormel J. The dyadic nature of bullying and victimization: testing a dual-perspective theory. Child Dev. 2007;78(6):1843–54. 10.1111/j.1467-8624.2007.01102.x. 17988325 10.1111/j.1467-8624.2007.01102.x

[13] Heydenberk RA, Heydenberk WR. Bullying reduction and subjective wellbeing: the benefits of reduced bullying reach Far beyond the victim. Int J Wellbeing. 2017;7(1):12–22. 10.5502/ijw.v7i1.516.

[14] Huitsing G, Lodder GMA, Oldenburg B, Schacter HL, Salmivalli C, Juvonen J, Veenstra R. The healthy context paradox: victims’ adjustment during an anti-bullying intervention. J Child Fam Stud. 2019;28(9):2499–509. 10.1007/s10826-018-1194-1.

[15] Laninga-Wijnen L, Yanagida T, Garandeau CF, Malamut ST, Veenstra R, Salmivalli C. Is there really a healthy context paradox for victims of bullying? A longitudinal test of bidirectional associations between victimization and psychological problems. Dev Psychopathol. 2025;37(1):40–54. 10.1017/S0954579423001384. 37990407 10.1017/S0954579423001384

[16] Zhang Y, Fang Y, Wang Y, Liu S, Wang X, Zhang S, Chen Z. Peer victimization and adolescent mental health: School-level victimization as a moderator. J Interpers Violence. 2024;39(21–22):4647–66. 10.1177/08862605241244473. 38587277 10.1177/08862605241244473

[17] Laninga-Wijnen L, van den Berg YHM, Mainhard T, Cillessen AHN. The role of defending norms in victims’ classroom climate perceptions and psychosocial maladjustment in secondary school. Res Child Adolesc Psychopathol. 2021;49(2):169–84. 10.1007/s10802-020-00738-0. 33301130 10.1007/s10802-020-00738-0 PMC7826303

[18] Yun HY, Juvonen J. Navigating the healthy context paradox: identifying classroom characteristics that improve the psychological adjustment of bullying victims. J Youth Adolesc. 2020;49(11):2203–13. 10.1007/s10964-020-01300-3. 32772331 10.1007/s10964-020-01300-3 PMC7538408

[19] Dijkstra JK, Gest SD. Peer norm salience for academic achievement, prosocial behavior, and bullying: implications for adolescent school experiences. J Early Adolescence. 2015;35(1):79–96. 10.1177/0272431614524303.

[20] Festinger L. A theory of social comparison processes. Hum Relat. 1954;7(2):117–40. 10.1177/001872675400700202.

[21] Sentse M, Scholte R, Salmivalli C, Voeten M. Person-group dissimilarity in involvement in bullying and its relation with social status. J Abnorm Child Psychol. 2007;35(6):1009–19. 10.1007/s10802-007-9150-3. 17588201 10.1007/s10802-007-9150-3

[22] Wright JC, Giammarino M, Parad HW. Social status in small groups: Individual-group similarity and the social misfit. J Personal Soc Psychol. 1986;50(3):523–36. 10.1037/0022-3514.50.3.523.

[23] Gong X, Huebner ES, Tian L. Bullying victimization and developmental trajectories of internalizing and externalizing problems: the moderating role of locus of control among children. Res Child Adolesc Psychopathol. 2021;49(3):351–66. 10.1007/s10802-020-00752-2. 33404945 10.1007/s10802-020-00752-2

[24] Schacter HL, White SJ, Chang VY, Juvonen J. Why me? Characterological self-blame and continued victimization in the first year of middle school. J Clin Child Adolesc Psychol. 2015;44(3):446–55. 10.1080/15374416.2013.865194. 24483145 10.1080/15374416.2013.865194 PMC6129379

[25] Garandeau CF, Vermande MM, Reijntjes AHA, Aarts E. Classroom bullying norms and peer status: effects on victim-oriented and bully-oriented defending. Int J Behav Dev. 2022;46(5):401–10. 10.1177/0165025419894722.

[26] Lodder GM, Scholte RH, Cillessen AH, Giletta M. Bully victimization: selection and influence within adolescent friendship networks and cliques. J Youth Adolesc. 2016;45(1):132–44. 10.1007/s10964-015-0343-8. 26323168 10.1007/s10964-015-0343-8 PMC4698289

[27] Hodges EVE, Perry DG. Personal and interpersonal antecedents and consequences of victimization by peers. J Personal Soc Psychol. 1999;76(4):677–85. 10.1037/0022-3514.76.4.677. 10.1037//0022-3514.76.4.677 10234851

[28] Sentse M, Dijkstra JK, Salmivalli C, Cillessen AH. The dynamics of friendships and victimization in adolescence: A longitudinal social network perspective. Aggressive Behav. 2013;39(3):229–38. 10.1002/ab.21469. 10.1002/ab.21469 23446945

[29] Cuadros O, Berger C. The protective role of friendship quality on the wellbeing of adolescents victimized by peers. J Youth Adolesc. 2016;45(9):1877–88. 10.1007/s10964-016-0504-4. 27230120 10.1007/s10964-016-0504-4

[30] Ladd GW, Kochenderfer BJ, Coleman CC. Classroom peer acceptance, friendship, and victimization: distinct relational systems that contribute uniquely to children’s school adjustment? Child Dev. 1997;68(6):1181–97. 10.2307/1132300. 9418233 10.1111/j.1467-8624.1997.tb01993.x

[31] Coie JD, Dodge KA, Coppotelli H. Dimensions and types of social status: A cross-age perspective. Dev Psychol. 1982;18(4):557–70. 10.1037/0012-1649.18.4.557.

[32] Gommans R, Cillessen AHN. Nominating under constraints: A systematic comparison of unlimited and limited peer nomination methodologies in elementary school. Int J Behav Dev. 2015;39(1):77–86. 10.1177/0165025414551761.

[33] Mérei F. Közösségek Rejtett hálózata. Osiris Kiadó; 1978.

[34] Olweus D. School bullying: development and some important challenges. Ann Rev Clin Psychol. 2013;9:751–80. 10.1146/annurev-clinpsy-050212-185516. 23297789 10.1146/annurev-clinpsy-050212-185516

[35] Sokolova V, Tomašević A, Dinić B, Jarić I. Evolution of students’ friendship networks: examining the influence of group size. Etnoantropološki Problemi / Issues Ethnology Anthropol. 2016;11(4):1135–51. 10.21301/eap.v11i4.10.

[36] Kulawiak PR, Wilbert J, Schlack R, Börnert-Ringleb M. Prediction of child and adolescent outcomes with broadband and narrowband dimensions of internalizing and externalizing behavior using the strengths and difficulties questionnaire. PLoS ONE. 2020;15(10). 10.1371/journal.pone.0240312. Article e0240312. 10.1371/journal.pone.0240312 PMC7546492 33035264

[37] McMullen JA, Veermans K, Laine K. Tools for the classroom? An examination of existing sociometric methods for teacher use. Scandinavian J Educational Res. 2014;58(5):624–38. 10.1080/00313831.2013.838694.

[38] Terry R. Recent advances in measurement theory and the use of sociometric techniques. In: Cillessen AHN, Bukowski WM, editors. Recent advances in the measurement of acceptance and rejection in the peer system. Jossey-Bass; 2000. pp. 27–53. 10.1002/cd.23220008805 10900969

[39] Gest SD, Graham-Bermann SA, Hartup WW. Peer experience: common and unique features of number of friendships, social network centrality, and sociometric status. Soc Dev. 2001;10:23–40. 10.1111/1467-9507.00146.

[40] Gifford-Smith ME, Brownell CA. Childhood peer relationships: social acceptance, friendships, and peer networks. J Sch Psychol. 2003;41(4):235–84. 10.1016/S0022-4405(03)00048-7.

[41] Hoffmann T, Basa B, Bernáth L, Kollár KN. The Role of Reciprocated Friendships in the Behavioral Correlates of SociometricCategories. *Canadian Journal of School Psychology*. 2024;40(1):21–39. https://journals.sagepub.com/doi/abs/10.1177/08295735241263807.

[42] Neal JW. Methodological moderators of average outdegree centrality: A meta-analysis of child and adolescent friendship networks. Netw Sci. 2024;12(2):107–21. 10.1017/nws.2024.2.

