# Supplementary material for: When healthy classrooms hurt: how reduced bullying isolates victimized youth through altered friendship and status dynamics
Source: BMC Psychol. 2026 Feb 18;14:410. doi: 10.1186/s40359-026-04141-0 (PMC13020164; doi:10.1186/s40359-026-04141-0)
Supplement: Supplementary file 1 — Supplementary Material 1. [file 40359_2026_4141_MOESM1_ESM.docx]

**Appendix**

English translation of bullying questions:
- Who in the class are those who, without any apparent reason, physically hurt, verbally abuse, or exclude others (either online or in person) who are unable to defend themselves? (Bully)

- Who in the class are those who, without any apparent reason, are physically hurt, verbally abused, or are excluded by others (either online or in person) and who are unable to defend themselves? (Victim)

- Who in the class disapprove of harrasment and harmful behaviors of others and try to make their classmates stop it? (Defender)
